# Supplementary material for: Repeatability, reproducibility, agreement, and safety of Tono-Pen tip cover for intraocular measurement using latex and polyethylene wrap
Source: PLoS One. 2020 Sep 28;15(9):e0239875. doi: 10.1371/journal.pone.0239875 (PMC7521710; doi:10.1371/journal.pone.0239875)
Supplement: S1 File — (PDF) [file pone.0239875.s001.pdf]

July 19, 2020

Re: Certificate of Service

Dear Editor-in-Chief:

I, Pirapon June Ohata, certify that Dr Krit Pongpirul has procured language proofreading and copyediting services offered through the Research Affairs at the Chulalongkorn University, Faculty of Medicine, for the manuscript entitled, “Repeatability, reproducibility, agreement, and safety of Tono-Pen tip cover for intraocular measurement using latex and polyethylene wrap” on July 15, 2020.

Please feel free to contact me at juneohata4@gmail.com should you have any questions pertaining to this matter.

Sincerely yours,

Pirapon June Ohata
